# Surgery

**Published:** 1860-09

**Authors:** 


### SURGERY.

1. ON COXO-FEMORAL DISARTICULATION IN RELATION TO MILITARY SURGERY. By *M. Legouest*.—After the battles of the Alma and of Inkermann this operation was performed upon thirteen patients, all of whom died. The case of one of these, a Russian prisoner, is related at some length by the author. He was very nearly being saved, when he had a fall upon his stump, and was carried off by diarrhœa, owing to his being placed amid a large assemblage of patients confined to a small space, many of whom were the subjects of scorbutus, hospital gangrene, or purulent infection, and almost all were suffering from diarrhœa, dysentery, or cholera. This approach to success has given the author little desire to repeat an operation the value of which, he says, is much less in the eyes of the surgeon than in those of the public; and he thinks its performance should be reserved for organic diseases of the femur incurable by any other means. He has endeavored to collect all the instances in which this operation has been performed after gunshot injury. In thirty of these the operation was *immediate*, the whole dying; in eleven it was *mediate*, eight dying and three recovering; and in three it was *ulterior*, two dying and one recovering. Of the first category, some died during the operation itself, others soon after they had been carried to their beds, and all within ten days, except two patients, related by Larrey, one of whom lived twenty-one and the other thirty days. Should not an operation furnishing such results be positively interdicted? The proportion of three recoveries in eleven mediate operations is, on the other hand, a large one, especially when we consider the mortality from amputation of the thigh, and probably arises from the fact of all the unsuccessful cases not having been made known. Ulterior operations—*i.e.* those performed about a year after the accident—have succeeded once in three times, analogous as they are to the amputation necessitated by disease of the femur. Now, can this slight amount of success, which is not even so great as it seems, be compared with the results derived from attempts at the preservation of the limb? Unfortunately, many surgeons, almost in spite of themselves, lay more stress upon a successful case of operation than upon a recovery obtained by assiduous care, and by an ingenious combination of all possible appliances, independently of cutting instruments. The success of a dangerous operation is willingly published, while the more humble but more precious triumphs of conservative surgery are unrecorded. This is the case with most of the instances of fracture of the cervix or the trochanters which have been treated with preservation of the limb. M. Legouest has, however, here collected six instances, one of these having occurred in Larrey's practice, another in Sédillot's practice, and four in his own. Of these six patients, three lived; while in forty-four cases of amputation of the thigh, recorded as having been performed by skillful surgeons, and from which all doubtful cases have been eliminated, scarcely can four cases of authentic cure be discovered.

The practical conclusions he arrives at are, first, that all immediate amputations—*i.e.* before the early phenomena of suppurative inflammation set in—should, as laid down by Sédillot in 1841, be rejected. At all events, every effort should be made to obtain some delay. When an operation has to be performed, on account of gunshot wound of the femur, without lesion of the vessels, with large splinters that cannot be extracted, an amputation through the trochanters, or immediately above them, is preferable to disarticulation. This operation does not deserve the proscription that has been directed against it. The wound is smaller and less deep, and the head of the femur remaining in the normal position, if it does not diminish the danger of the operation, facilitates the subsequent application of prothetic apparatus. When, too, there is intra-articular fracture of the cervix, or fracture of the head of the bone itself, resection of the upper part of the femur should be preferred to disarticulation. This operation has now been performed in twelve or fourteen cases, and in more than a third it has succeeded. It is true that it failed in the two cases of gunshot fracture it was tried in; but how many times had coxo-femoral disarticulation been attempted before four authentic recoveries could be recorded? Complete ablation of the limb should be reserved for cases of fracture with lesion of vessels, for if these remained intact, resection should be preferred. To sum up: in ordinary cases—which are by far the most numerous—we shall derive more advantage from preserving than from removing the limb; and when an operation is determined upon, we shall defer it to the latest period consistently with the general condition of the patient.—(*British and Foreign Medico-Chirurgical Review*, January, 1860.)

2. REPORT ON THE CONDITION OF THE PROSTATE IN OLD AGE, FOUNDED ON THE DISSECTION OF ONE HUNDRED SPECIMENS IN INDIVIDUALS OVER SIXTY YEARS OF AGE. By *John Cockburn Messer, M.D., R.N.*, Assistant Surgeon to the Royal Hospital, Greenwich.—In order to facilitate the consideration of the details of one hundred dissections of the prostate after the age of sixty, the author had arranged them into three classes, namely—

First. Those under four drachms weight.

Second. Those between four drachms and six drachms weight.

Third. Those over six drachms weight.

By so doing a broad division is at once made between those that are comparatively healthy—namely, the first and second classes—and those that are so altered as to be likely to affect the health of the patient, comprised in the third class.

In the first class there are twenty cases, giving—

| | Minimum. | Maximum. | Medium. |
|---|---|---|---|
| Age | 60 | 94 | 76·2 |
| Weight | 4 drachms. | 6 drachms. | 4 drachms 57 grains. |

These cases, for the most part, differed from the normal state only in point of size, and offered no obstruction to the flow of urine. The presence of small black concretions was very general in these as well as in all the other cases. In four cases there were slight appearances of the formation of circumscribed tumors. In one case abscess was found associated with stricture of the urethra.

In one the posterior lobe showed a tendency to enlargement; but it was difficult to say whether the enlargement was more intimately connected with the prostate or with a fasciculus of the muscular coat of the bladder.

In the second class are forty-five cases, which may be considered normal in condition, and which give—

| | Minimum. | Maximum. | Medium. |
|---|---|---|---|
| Age | 60 | 94 | 76·2 |
| Weight | 4 drachms. | 6 drachms. | 4 drachms 55 grains. |

None of these cases suffered from urinary obstruction connected with the prostate during life, although the bladder was often found fasciculated. In twelve of these, circumscribed tumors were observed, for the most part only slightly developed; in three, the posterior lobe was slightly enlarged; in one, abscess was present, the consequence of general paralysis.

In the third class are thirty-five cases, which give—

| | Minimum. | Maximum. | Medium. |
|---|---|---|---|
| Age | 60 | 87 | 75·2 |
| Weight | 6 drachms 15 grains. | 48 drachms. | 15 drachms 2 grains. |

In seventeen of these, the enlargement affected both lateral and posterior lobes; in fourteen, the enlargement existed chiefly in both lateral lobes; in one, the enlargement affected only the left lateral and posterior lobes; in one, enlargement preponderated in the left lateral and posterior lobes; in one, enlargement preponderated in the left lateral lobe; in one, enlargement preponderated in the posterior lobe. Thus it appears that 35 per cent. of all prostates after the age of sixty are abnormally large, 20 per cent. are abnormally small, and 45 per cent. are within the limits of the normal weight. This enlargement is principally caused by increase of the fibrous element of the body; the glandular also being increased in amount, but not to the same degree. This new fibrous tissue is deposited in concentric layers, and so forms circumscribed tumors. The frequency of this fibrous deposit is shown by the fact that it was present in thirty-four out of thirty-five cases of enlargement, in twenty-seven of which it was found in the form of tumors; in seven there was no appearance of tumors. It also appears that those glands in which the tumors are marked are liable to the greatest enlargement, as some thus affected were found to weigh thirty drachms, and even forty-eight drachms, while those in which the tumors did not appear never weighed more than seventeen drachms.

A comparison of the relative frequency of enlargement of the different parts of the gland shows that the lateral lobes are much more liable to be affected than the posterior: thirty-four of thirty-five cases were affected in their lateral lobes, while only nineteen of the same number were affected in the posterior lobe. It is rare to find the posterior lobe enlarged while the rest of the gland is normal; only one such case in thirty-five was found. Enlargement of the posterior lobe is the chief cause of obstruction to the flow of urine; but that may also be the consequence of hypertrophy of the lateral lobes, especially when it takes the form of tumors, and they project inward upon the urethra.

It appears, from the nearly equal average age in all three classes, that the condition of the prostate does not materially affect the longevity of the individual. A slight difference does, however, exist in favor of those in whom the gland is most nearly normal, the average in these being 76·2, and in the enlarged 75·2.

The presence of abscess in the prostate produces enlargement to a greater or less extent, seldom, however, to the same extent as fibrous deposit. The most frequent cause of abscess in the prostate appears to be obstruction to the flow of urine, either from stricture of the urethra, enlargement of the prostate, or the consequence of paralysis of the bladder. The frequency of abscess in the enlarged gland is in the proportion of five in thirty-five; in those between four drachms and six drachms, one in forty-five; in those under four drachms, one in twenty. The causes in these cases were—stricture of urethra in three cases; frequent retention in three cases; paralysis of bladder in one case. Tubercle is the only other abnormal deposit giving rise to enlargement of the prostate noticed in these cases, and that only in one case, which weighed twenty-four drachms. A similar deposit was observed in the lungs, right kidney, and mucous coat of the bladder in this subject.

It is worthy of remark that while retention of urine, more or less complete, is the most important symptom and consequence of enlarged prostate, it is not found in every case. The proportion of men in advanced years suffering from the consequences of enlarged prostate is indeed small. Thus among sixteen hundred old men, with an average sick list of two hundred, not more than ten are under treatment for this disease, and half of these only occasionally. A much larger number must be affected with enlargement, as shown by post-mortem examinations of the gland. In thirty-five cases of enlargement found after death, thirteen suffered no urinary symptoms during life, and two others only after the occurrence of serious leisons to the nervous system shortly before death. Although many of these cases were not greatly enlarged, some of them plainly showed that the prostate may be greatly altered, and yet the patient be free from urinary obstruction, as in one case, where the prostate weighed eight drachms thirty grains, with prominent enlargement of the posterior lobe; in another, which weighed nineteen drachms thirty grains, with general hypertrophy and great encroachment on the urethra; in another, which weighed twenty-six drachms thirty grains, with the enlargement principally seated in the lateral lobes.

On considering the favorable circumstances for the formation of phosphatic calculi in cases of enlarged prostate, it is surprising that these concretions are not more frequently found. Of the thirty-five cases of the third class, phosphatic calculi were found in two, the largest weighing seven drachms forty-five grains; in another, two uric acid calculi, of about thirty grains each, were found.—(*London Lancet*, May 19, 1860.)

